# Performance of femtosecond laser-assisted cataract surgery in Chinese patients with cataract: a prospective, multicenter, registry study

**DOI:** 10.1186/s12886-019-1079-0

**Published:** 2019-03-14

**Authors:** Xiaobo Zhang, Yinhui Yu, Guangbin Zhang, Yanwen Zhou, Guangyu Zhao, Maosheng Chen, Yong Wang, Siquan Zhu, Hong Zhang, Ke Yao

**Affiliations:** 1 Eye Center, Second Affiliated Hospital, School of Medicine, Zhejiang University, Hangzhou, China; 2 Cataract, Xiamen Eye Center, Xiamen, China; 3 Cataract, Shenyang Aier Eye Hospital, Shenyang, China; 4 Cataract, Fuzhou Southeast Ophthalmology Hospital, Fuzhou, China; 5 Cataract, Chongqing Aier Ophthalmology Hospital, Chongqing, China; 6 Cataract, Wuhan Aier Eye Hospital, Wuhan, China; 7 Ophthalmology, Beijing Tongren Hospital, Beijing, China; 8 Cataract, Ophthalmology Hospital of Tianjin Medical College, Tianjin, China

**Keywords:** Femtosecond laser–assisted cataract surgery, Intraoperative complication, Chinese patients

## Abstract

**Background:**

This study aimed to investigate the completion rate, visual performance, and adverse outcomes of femtosecond laser-assisted cataract surgery (FLACS) in Chinese patients.

**Methods:**

This is a prospective, single-arm, multicenter registry study of 19 cataract surgery clinics in China. Chinese patients with cataract who underwent FLACS using the Alcon LenSx® laser system in single eye (*n* = 1140) or both eyes (*n* = 201) were enrolled and data were collected between March 2015 and August 2016. Clinical characteristics were recorded before surgery, and on postoperative days 1, 7, and 30. For surgery on both eyes, the second eye was included in the analysis only if it was operated within 30 days after the first eye surgery. The primary outcome was the completion rate of circular anterior capsulotomy. Secondary outcomes for lens fragmentation, corneal incision, and intraocular lens (IOL) implantation included best corrected distance visual acuity (BCDVA) and completion rates. Adverse events (AEs) were recorded.

**Results:**

The completion rates of circular anterior capsulotomy, lens fragmentation, corneal incision, and IOL implantation were 98.6% (95% CI: 97.8–99.1%), 99.5% (95% CI: 99.1–99.8%), 97.6% (95% CI: 96.7–98.3%), and 100% (95% CI: 99.8–100%), respectively. BCDVA preoperatively and at postoperative day 30 were 1.134 ± 0.831 logMAR and 0.158 ± 0.291 logMAR, respectively. The proportion of eyes with BCDVA of 20/20 or better was 1.6% at baseline and 41.3% at postoperative day 30. AE incidence was 0.32%, with posterior capsule rupture present in 0.19% of eyes.

**Conclusion:**

FLACS using the LenSx® laser system can achieve satisfactory results in a real-world setting.

**Electronic supplementary material:**

The online version of this article (10.1186/s12886-019-1079-0) contains supplementary material, which is available to authorized users.

## Background

Cataract is a major global health problem and the most common underlying cause of reversible blindness worldwide [1, 2]. In 2010, cataract was responsible for blindness in nearly 11 million people and visual impairment in a further 35 million people [3]. Socioeconomic factors are known to influence cataract prevalence and management [4], and developing countries have a higher prevalence of blindness due to cataract [3]. In China, cataract is the major cause of blindness, and the prevalence of age-related cataract has been estimated to be 38.1% [5].

The management of cataract involves surgery and cataract operations are amongst the most common surgical procedures performed worldwide, although cataract surgery rates (CSR) vary widely between developed and developing countries [6]. The conventional technique involves the use of a small incision plus phacoemulsification to remove the lens material followed by the insertion of an intraocular lens (IOL). In this method, the ophthalmic surgeon opens the anterior lens capsule with surgical instruments, a procedure known as continuous curvilinear capsulorhexis (CCC). In recent years, there has been an increase in the use of femtosecond laser-assisted cataract surgery (FLACS), which has been reported to have several advantages over conventional methods with regard to opening of the anterior capsule [7, 8]. Several studies have found that FLACS creates circular, smooth and rupture-resistant capsulotomies [9] that are at least as strong if not stronger than manual capsulorhexis [10]. FLACS can achieve capsulotomies that are more circular and more accurate in size than manual capsulorhexis [11, 12]. Furthermore, FLACS has been reported to result in better IOL centration than manual capsulotomy [13]. Another recognized benefit of FLACS compared to the conventional technique is the reduction in phacoemulsification time and cumulative dissipation energy [14, 15]. In addition, there are some evidences that recovery of visual acuity is faster with FLACS than manual capsulorhexis [12, 16]. Importantly, FLACS is considered to be associated with a similar or perhaps lower incidence of intra-operative and post-operative complications than conventional phacoemulsification [17, 18]. Moreover, most investigations indicate that changes in corneal endothelial cell density, corneal thickness and/or central retinal thickness are similar or smaller for FLACS than for manual capsulorhexis [19–21].

Nevertheless, there are limited ‘real-world’ data regarding the performance of FLACS in the management of cataract. Real-world data have comprehensive guiding significance in objective evaluation of the novel technique. Therefore, the aim of the present study was to investigate the performance of the Alcon LenSx® laser system in real-world medical practice.

## Methods

### Study design and patients

This was a prospective, single-arm, multicenter, registry study. Chinese patients with cataract who underwent FLACS in 19 cataract surgery clinics in China (Table 1) using the LenSx® laser system in at least one eye between March 2015 and August 2016 were enrolled. The inclusion criteria were: 1) patients with age-related cataract or complicated cataract; 2) > 18 years of age; 3) planned to receive LenSx® laser-assisted cataract surgery and phacoemulsification in at least one eye; and 4) written informed consent was provided by the patient prior to enrolment in the registry. The exclusion criteria were: 1) had contraindication to FLACS, as listed in the LenSx® laser system operator’s manual (e.g. pupils dilation of < 5.0 mm, small palpebral fissure, nystagmus, severe conjunctivochalasis, small hyperopic eyes with steep cornea (hard for suction), dense cornea scars and edema, uncooperative patient, or a functioning bleb, tube, or valve); 2) women who were pregnant, nursing or planning a pregnancy; 3) participated in other clinical trials before enrollment, or did not cooperate in the examinations, or did not adhere to the follow-up; or 4) had any other conditions that warranted exclusion based on the physician’s judgment. This study was approved by the ethics committees of all the participating centers (Additional file 1) and adhered to the principles of the Declaration of Helsinki. Patients were withdrawn from the study if serious intraoperative complication occurred, including loss of vitreous, hyphema, intraocular hypertension, capsule rupture, endophthalmitis, conjunctivitis, or infectious keratitis. Their data would be captured in adverse event (AE) reports.

**Table 1:** Number of subjects treated by investigator site

Number	Site	Enrolled subjects (*N* = 1341), *n* (%)
01	The 2nd Affiliated Hospital of ZheJiang University	190 (14.2)
02	The 4th Affiliated Hospital of China Medical University	67 (5.0)
03	Tianjin Medical University Eye Hospital	90 (6.7)
04	Shanxi Eye Hospital	38 (2.8)
05	Jinan Mingshui Eye Hospital	63 (4.7)
07	ChiaMan Eye Hospital	114 (8.5)
08	Chongqing Aier Eye Hospital	102 (7.6)
09	Fuzhou South East Eye Hospital	92 (6.9)
10	Wuhan Aier Eye Hospital	108 (8.1)
11	Yinzhou Eye Hospital	22 (1.6)
12	The 180th Hospital of PLA	47 (3.5)
13	Weifang Eye Hospital	71 (5.3)
14	Shandong Shi E Ming Eye Hospital	37 (2.8)
15	Beijing Tongren Hospital, Capital Medical University	91 (6.8)
16	Shenyang Aier Eye Hospital	131 (9.8)
17	Mianyang Center Hospital	46 (3.4)
18	Nanjing South East Eye Hospital	12 (0.9)
19	The Eye Hospital of WMU - Hangzhou	16 (1.2)
21	The 2nd Affiliated Hospital of Dalian Medical university	4 (0.3)

### Baseline data collection

The following baseline characteristics were recorded at a screening visit (1–30 days before surgery) or on the day of surgery: age, sex, ethnicity, uncorrected distance visual acuity (UCDVA, logarithm of the minimal angle of resolution [logMAR]), best corrected distance visual acuity (BCDVA, logMAR and Snellen Chart), maximum keratometry reading (Kmax, D) and minimum keratometry reading (Kmin, D), axial length (mm), nuclear density with the Emery-Little classification (soft, semi-soft, semi-hard, hard or very hard) [22], endothelial cell count (per mm^2^), and pupil status. Visual acuity was assessed with a standard logarithm visual acuity chart and Snellen Chart. Keratometry was assessed with an IOL-Master 500 (Carl Zeiss Meditec AG, Iena, Germany), a LenSTAR LS 900 (Haag-Streit AG, Koeniz, Switzerland), or a Scheimpflug imaging system (Pentacam, OCULUS Optikgerate GmbH, Wetzlar, Germany). Axial length was measured with an IOL-Master 500 (Carl Zeiss Meditec AG, Iena, Germany) or a LenSTAR LS 900 (Haag-Streit, Koeniz, Switzerland). When the lens was too opaque for measurement, A-scan (A-mode US, Cinescan, Quantel Medical, Cournon-d’Auvergne, France) was used.

### Surgical technique

All surgeons from 19 cataract surgery clinics received unified surgical training at the Second Affiliated Hospital, School of Medicine, Zhejiang University, Hangzhou, China before joining the study, and the surgical procedures followed our previous study [23] using the LenSx® laser system (Alcon, Fort Worth, TX, USA). This system was the first commercially available system for laser cataract surgery, which contains an optical coherence tomography (OCT) system. It uses a disposable curved patient interface for docking and live OCT imaging. The femtosecond laser was used for the capsulotomy, lens fragmentation, and corneal incisions before phacoemulsification. The operated eye was applanated with the disposable interface contact lens of the femtosecond laser system using the suction ring supplied by the manufacturer (SoftFit Patient Interface, Alcon-LenSx, Inc.). The proprietary energy and spot separation parameters, which had been optimized in previous studies, were used for all procedures. All surgical procedures were performed using standard equipment and by experienced surgeons with an experience of at least 50 FLACS before this study. Postoperative medications were identical for all operated eyes, same as our previous study [23], which consisted of topical dexamethasone tobramycin 4 times a day for 2 weeks and pranoprofen 4 times a day for 1 month.

### Outcome assessment

The primary outcome was the completion rate of a circular anterior capsulotomy. The completion of a circular anterior capsulotomy was defined as complete capsular edge separation being achieved and no manual separation of residual tags is needed. The secondary outcomes were the completion rates of FLACS procedures including lens fragmentation, corneal incision, and successful IOL implantation, as well as the result of BCDVA. If the surgeons observed such situations including but not limited to broken pieces, fragmentation trace of lens nucleus or retrolenticular bubbles after laser lens fragmentation, it would be judged as lens fragmentation completion. The completion of corneal incisions was defined as a blunt spatula can be used to open the laser-incised cornea. The completion of IOL implantation was defined as IOL implantation into the eye successfully during the FLACS. All patients were followed up postoperatively at 1 day (±1 day), 7 days (±2 day) and 30 days (±14 days) regardless of whether the surgery was for the first or second eye. If cataract surgeries were performed in both eyes, the patient was followed up until postoperative day 30 post the surgery of the second eye.

### Safety analysis

All AEs, whether or not related to the investigational product, were collected, recorded, and reported. Serious AEs (SAEs) were defined as AEs that led to: 1) death; 2) any serious deterioration in the condition of the patient; 3) any life-threatening event; 4) any potentially sight-threatening event or permanent impairment to any body structure/function; 5) prolonged hospitalization or new event of hospitalization; 6) any harm due to false positive or false negative result from a diagnostic test that was used within the manufacturer’s indications and instructions; and 7) any event leading to threats to normal fetal development and birth.

In addition to reporting all AEs (serious and non-serious) meeting the definitions, the investigators had to report any occurrence of the following as an SAE in this study: capsular tear, vitreous loss, anterior segment hyphema, anterior chamber collapse and iris damage. Treatment emergent AEs (TEAEs) were defined as AEs after study treatment which referred to FLACS in this study.

### Statistical analysis

Assuming a completion rate of a circular anterior capsulotomy of 92%, an α of 0.05 and a power of 0.8, a sample size of 1500 eyes was estimated to be sufficient to ensure that a precise estimate of the 95% confidence interval (CI) would fall within a rate of ±1.5%. The data were analyzed using descriptive statistics. Continuous variables were expressed as means ± standard deviation (SD) or medians (range). Categorical variables were expressed as frequencies and percentages, and compared using the chi-square test or continuity correction X^2^ test. All statistical analyses were performed using SPSS 21.0 (IBM, Armonk, NY, USA). *P* value < 0.05 was considered statistically significant.

## Results

### Baseline characteristics

**Fig. 1:**
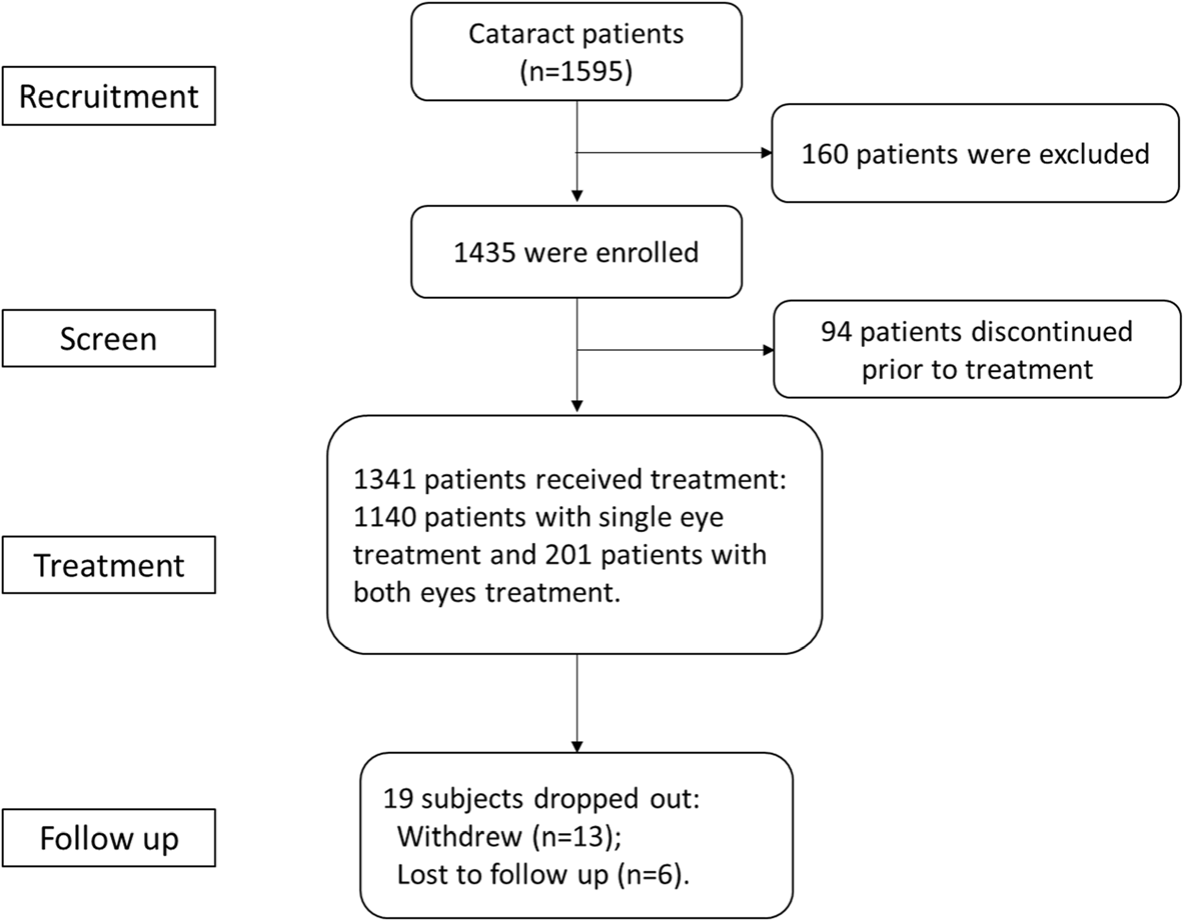
Patient flowchart

A total of 1435 patients were enrolled in 19 cataract surgery clinics in China, and 1341 patients (males: 669; females: 672) were finally treated (Table 1). Among the patients who dropped out (*n* = 19), 13 patients withdrew consent and six were lost to follow-up (Fig. 1). A total of 1542 eyes were included in the final analysis (data for single eye treatment in 1140 patients and for both eyes treatment in 201 patients). The demographic characteristics of patients are presented in Table 2. The mean values of UCDVA, BCDVA, Kmax, and Kmin for all treated eyes were 1.134 ± 0.831 logMAR, 0.878 ± 0.854 logMAR, 44.52 ± 1.81 D, and 43.55 ± 1.78 D, respectively. The average axial length of all treated eyes was 24.53 ± 2.54 mm. Preoperative nuclear density for all treated eyes was 2.8 ± 0.77, and the majority of eyes (51.3%) were classified as having a semi-hard nuclear density. The endothelial cell count at baseline was 2600 ± 371/mm^2^ for all treated eyes (Table 3). The spherical power and cylinder power were 4.64 ± 5.42 and 1.36 ± 1.09 for all treated eyes at baseline.

**Table 2:** Demographic characteristics of the patients

Characteristic	Total (*n* = 1341)
Age (years)	64.8 ± 13.4
Sex, *n* (%)	
Male	669 (49.9)
Female	672 (50.1)
Ethnicity, *n* (%)	
Han	1327 (99.0)
Others	14 (1.0)

Data are shown as mean ± standard deviation, unless otherwise stated

**Table 3:** Baseline characteristics of eyes

Characteristic	Total (*n* = 1542)
UCDVA (logMAR)	1.134 ± 0.831
BCDVA (logMAR)	0.878 ± 0.854
Kmax (Diopters)	44.52 ± 1.81
Kmin (Diopter)	43.55 ± 1.78
Axial length (mm)	24.53 ± 2.54
Nuclear density, *n* (%)	
Soft	51 (3.3)
Semi-soft	448 (29.0)
Semi-hard	791 (51.3)
Hard	233 (15.1)
Very hard	18 (1.2)
Missing data	1 (0.1)
Endothelial cell count (/mm^2^)	2600 ± 371

Data are shown as mean ± standard deviation, unless otherwise stated. *BCDVA* best corrected distance visual acuity, *Kmax* maximum keratometry reading, *Kmin* minimum keratometry reading, *UCDVA* uncorrected distance visual acuity

### Primary and secondary outcomes

According to the results of primary and secondary outcome measures shown in Tables 4 and 5, the completion rate of a circular anterior capsulotomy was 98.6% (95% CI: 97.8, 99.1%) for all 1542 treated eyes. The completion rate of lens fragmentation was 99.5% (95% CI: 99.1, 99.8%) for all treated eyes. Fragmentation was successfully completed for all lenses categorized as soft or semi-soft; the overall lens fragmentation completion rates for lenses with semi-hard, hard and very hard nuclei were 99.5, 99.1 and 94.4%, respectively. The completion rate of corneal incision was 97.6% (95% CI: 96.7, 98.3%) overall. The completion rate of IOL implantation was 100% (95% CI: 99.8, 100%) for all treated eyes. Baseline BCDVA was 0.887 ± 0.861 logMAR. Improvement occurred postoperatively with BCDVA 0.186 ± 0.324 logMAR at postoperative day 7 and 0.158 ± 0.291 logMAR at postoperative day 30 at the follow-up points. The proportion of all treated eyes with a BCDVA of 20/20 or better was 1.6% at baseline, 36.5% at postoperative day 7 and 41.3% at postoperative day 30. The proportion of all treated eyes with a BCDVA worse than 20/40 was 74.1% at baseline, 15.2% at postoperative day 7, and 12.6% at postoperative day 30.

**Table 4:** Primary and secondary outcomes

Variable	Total (*n* = 1542)
Completion of a circular anterior capsulotomy	
*n* (%)	1520 (98.6)
95% CI	97.8, 99.1
Completion of lens fragmentation	
*n* (%)	1535 (99.5)
95% CI	99.1, 99.8
Completion of corneal incision	
*n* (%)	1505 (97.6)
95% CI	96.7, 98.3
Completion of IOL implantation	
*n* (%)	1542 (100)
95% CI	99.8, 100

*CI* confidence interval, *IOL* intra-ocular lens

**Table 5:** Best corrected distance visual acuity (BCDVA)

BCDVA (Snellen Chart)	Total (*n* = 1542)
Baseline	
*n*	1527
20/20 or better	24 (1.6)
20/25	77 (5.0)
20/32	31 (2.0)
20/40	264 (17.3)
Worse than 20/40	1131 (74.1)
Day 7	
*n*	1527
20/20 or better	557 (36.5)
20/25	364 (23.8)
20/32	50 (3.3)
20/40	324 (21.2)
Worse than 20/40	232 (15.2)
Day 30	
*n*	1520
20/20 or better	628 (41.3)
20/25	379 (24.9)
20/32	37 (2.4)
20/40	285 (18.8)
Worse than 20/40	191 (12.6)

Data are presented as *n* (%)

### Safety

One pre-treatment AE of hypertension was reported. TEAEs occurred in five treated eyes (an incidence of 0.32%), whereas no treatment-emergent non-ocular AEs was reported. The most frequent TEAE was miosis (nine cases, 0.58%), which was defined as an obvious pupil diameter decrease that influence the following surgical procedure. All these cases were of mild severity and considered related to the investigational product. The second most frequent TEAE was posterior capsule rupture (three cases, 0.19%), which was considered as a SAE. All three cases of posterior capsule rupture were of moderate severity; two were considered not related to the investigational product or procedure while one was deemed related to the investigational product. Lens fragmentation was completed for all three patients with posterior capsule rupture. Phaco surgery and IOL implantation were all completed in these three patients and no patients discontinued from the study because of AEs. Other TEAEs included vertigo (one case, 0.064%), which was of mild severity and considered not related to the investigational product or procedure.

## Discussion

FLACS is becoming increasingly used in the management of cataract, and several recent meta-analyses have identified advantages of FLACS over manual phacoemulsification including improved capsulotomy quality, reduced effective phaco time and energy, smaller changes in central corneal thickness, less corneal endothelial cell loss, and better visual outcomes in the short-term [24, 25]. The present study was designed to investigate the performance of the LenSx® laser system in real-world medical practice in Chinese patients with cataract. The main findings were that completion of a circular capsulotomy, lens fragmentation, IOL implantation, and corneal incision were successfully achieved in the vast majority of patients. Furthermore, the majority of treated eyes (66.2%) had a BCDVA of 20/25 or better at postoperative day 30, compared with only 6.6% at baseline; only 12.6% of treated eyes had a BCDVA worse than 20/40. In addition, AEs were reported in only 0.32% of cases, and the incidence of posterior capsule rupture was only 0.19%. These real-world clinical data in a large sample of patients support the conclusions of previous studies that FLACS is an effective and safe technique for the treatment of cataract.

The use of the LenSx® laser system in the present study achieved a 98.6% completion rate for circular capsulotomy. This is similar to the results of a previous study of the LenSx® laser system in Japanese eyes (98.7%) [26]. In this study, complications were seen in only 0.32% of patients, and posterior capsule rupture occurred in only 0.19% of patients. This could be due to more circular and precisely sized capsulotomies using FLACS than when using manual capsulorhexis [12, 27]. In addition, the completion of a circular anterior capsulotomies was defined as achieving complete capsular edge separation without manual separation of residual tags during surgery. This broad definition may have improved our completion rate. The completion rate for lens fragmentation (99.5%) was high and the lens fragmentation completion rates for lenses with semi-hard, hard, and very hard nuclei were 99.5, 99.1, and 94.4%. The completion rate of corneal incision (97.6%) was also very high. The high completion rate may be attributed to the rich experience of the investigators and strict inclusion criteria. In this study enrollment did not include patients with any contraindications to FLACS such as white cortical cataracts with a hard nucleus, corneal disease and dense corneal scar, in which a femtosecond laser could not be used for lens fragmentation or corneal incision. The completion rate of IOL implantation was 100% in our study. Previous study also suggested that FLACS can obtain better IOL centration than manual capsulotomy [12, 13], indicating a possible advantage of FLACS over conventional methods.

Visual outcomes are the primary concern for patients having cataract surgery. In this study, most patients achieved improved UCDVA and BCDVA postoperatively. The average value of BCDVA was 0.887 logMAR at baseline and 0.158 logMAR at postoperative day 30, indicating a substantial improvement. Furthermore, before surgery, 74.1% of patients had a BCDVA worse than 20/40, and only 1.6% had a BCDVA of 20/20 or better. By contrast, the corresponding values 30 days after FLACS were 12.6 and 41.3%, respectively. Our data are broadly in agreement with other published studies reporting improvements in BCDVA to between 0.004 and 0.89 logMAR at 1–3 months after FLACS [28] or in UCDVA to 0.12logMAR [29]. Other studies have reported that after FLACS, 97.5% of patients achieved a BCDVA of 20/40 or better [30] which were consistent with the present results. It is also notable in our study that the BCDVA data at postoperative day 7 (BCDVA of 0.186 ± 0.324 logMAR with 36.5% of patients obtained a BCDVA of 20/20 or better and 15.2% worse than 20/40) were broadly similar to those at postoperative day 30, suggesting that recovery of visual acuity after FLACS was rapid.

In this study, the most frequent intraoperative complication of FLACS was miosis (0.58%). In a previous study, Manning et al. reported eight cases of miosis in 2814 (0.3%) femtosecond-assisted cases, and 60 (1.2%) miosis in 4987 conventional phacoemulsification cases. They also demonstrated that the lower rate of miosis in the FLACS group may be related to that FLACS keeps a 1000-μm distance between the iris and capsule to prevent iris damage and subsequent intraoperative miosis [28]. In this study, there were three cases of reported posterior capsule rupture as SAE, which occurred in the process of phacoemulsification. Among the three cases, two patients underwent anterior vitrectomy. All patients with posterior capsule rupture were implanted IOL successfully and the postoperative vision recovered well. In 2015, Chen reported two cases of inadvertent opening in the posterior capsule when the LenSx Laser was used on 273 eyes. They did not find the exact cause but the author emphasized the importance of proper training to ensure the safety and the effect of FLACS [18].

This study has several limitations. First, this was a single-arm registry study, hence comparisons of outcomes and complications between FLACS and manual phacoemulsification were not undertaken. The inclusion criteria did not specify the baseline BCDVA and comorbidity, which might result in a low proportion of patients achieving 20/20. Second, although this was a multi-center study, the majority of patients were of Han origin, so the generalizability of these findings to other ethnicities remains unknown. Third, detailed analyses of data for additional visual outcomes such as UCDVA and mean absolute error of refraction were not performed. Fourth, measurement of phaco time and energy, endothelial cell density, central corneal thickness, central retinal thickness and IOL centration were not included as assessed parameters. Further studies are needed to confirm and extend our findings.

## Conclusions

The present registry study indicated that FLACS using the LenSx® laser system can achieve satisfactory results in a real-world setting. Our analysis of real-world clinical data from Chinese patients with cataract suggests that FLACS using the LenSx® laser system is an effective and safe technique for the surgical management of cataract.

## Additional file

Additional file 1: Participating centers. This file includes the name of participating centers, region, the number of enrolled patients at each center, and the approval numbers. (DOCX 18 kb)

## Abbreviations

AE: adverse event
BCDVA: best corrected distance visual acuity
CCC: continuous curvilinear capsulorhexis
CI: confidence interval
CSR: cataract surgery rates
FLACS: femtosecond laser-assisted cataract surgery
IOL: intraocular lens
LogMAR: logarithm of the minimal angle of resolution
OCT: optical coherence tomography
SAE: serious adverse event
SD: standard deviation
TEAE: treatment emergent adverse event
UCDVA: uncorrected distance visual acuity
