# Supplementary material for: Performance of femtosecond laser-assisted cataract surgery in Chinese patients with cataract: a prospective, multicenter, registry study
Source: BMC Ophthalmol. 2019 Mar 14;19:77. doi: 10.1186/s12886-019-1079-0 (PMC6417229; doi:10.1186/s12886-019-1079-0)
Supplement: Supplementary file 1 — Participating centers. This file includes the name of participating centers, region, the number of enrolled patients at each center, and the approval numbers. (DOCX 18 kb) [file 12886_2019_1079_MOESM1_ESM.docx]

**Additional file 1. Participating centers**

| Number | Center | Region | Enrolled subjects (N=1341), *n* (%) | Ethics committee | Approval number^*^ |
| --- | --- | --- | --- | --- | --- |
| 1 | The 2nd Affiliated Hospital of Zhejiang University | Hangzhou, Zhejiang province, China | 190 (14.2) | The 2nd Affiliated Hospital of Zhejiang University Ethics Committee | 2015-05-Q-2 |
| 2 | The 4th Affiliated Hospital of China Medical University | Shenyang, Liaoning province, China | 67 (5.0) | The 4th Affiliated Hospital of China Medical University Ethics Committee | 2015-001 |
| 3 | Tianjin Medical University Eye Hospital | Tianjin, China | 90 (6.7) | Tianjin Medical University Eye Hospital Ethics Committee | 201502 |
| 4 | Shanxi Eye Hospital | Taiyuan, Shanxi province, China | 38 (2.8) | ShanXi Eye Hospital Ethics Committee | - |
| 5 | Jinan Mingshui Eye Hospital | Jinan, Shandong province, China | 63 (4.7) | Jinan Mingshui Eye Hospital Ethics Committee | (2015) Medicine No.1 |
| 7 | ChiaMan Eye Hospital | Xiamen, Fujian province, China | 114 (8.5) | ChiaMan Eye Hospital Ethics Committee | - |
| 8 | Chongqing Aier Eye Hospital | Chongqing, China | 102 (7.6) | Chongqing Aier Eye Hospital Ethics Committee | (2015)-(3) |
| 9 | Fuzhou South East Eye Hospital | Fuzhou, Fujian province, China | 92 (6.9) | Fuzhou South East Eye Hospital Ethics Committee | 2015-001 |
| 10 | Wuhan Aier Eye Hospital | Wuhan, Hubei province, China | 108 (8.1) | Wuhan Aier Eye Hospital Ethics Committee | 2015IRBQX01 |
| 11 | Yinzhou Eye Hospital | Ningbo, Zhejiang province, China | 22 (1.6) | Yinzhou Eye Hospital Ethics Committee | - |
| 12 | The 180th Hospital of PLA | Quanzhou, Fujian province, China | 47 (3.5) | The 180th Hospital of PLA Ethics Committee | (2015)-(001) |
| 13 | Weifang Eye Hospital | Weifang, Shandong province, China | 71 (5.3) | Weifang Eye Hospital Ethics Committee | (2015) Medicine No.2 |
| 14 | Shandong Shi E Ming Eye Hospital | Jinan, Shandong province, China | 37 (2.8) | Shandong Traditional Chinese Medical College Eye Research Institute Ethics Committee | CTO130-P001 |
| 15 | Beijing Tongren Hospital, Capital Medical University | Beijing, China | 91 (6.8) | Capital Medical University Beijing Tongren Hospital Ethics Committee | TREC2015-16 |
| 16 | Shenyang Aier eye Hospital | Shenyang, Liaoning province, China | 131 (9.8) | Shenyang Aier eye Hospital Ethics Committee | - |
| 17 | Mianyang Center Hospital | Mianyang, Sichuan province, China | 46 (3.4) | Mianyang Center Hospital Ethics Committee | S2015010 |
| 18 | Nanjing South East Eye Hospital | Nanjing, Jiangsu province, China | 12 (0.9) | Nanjing South East Eye Hospital Ethics Committee | (2015) Medicine No.01 |
| 19 | The Eye Hospital of WMU - Hangzhou | Hangzhou, Zhejiang province, China | 16 (1.2) | The Eye Hospital of WMU Ethics Committee | CTO130-P001 |
| 21 | The 2nd Affiliated Hospital of Dalian Medical University | Dalian, Liaoning province, China | 4 (0.3) | The 2nd Affiliated Hospital of Dalian Medical University Ethics Committee | 2015-96 |

*Four ethics committees do not use an approval numbering system, but they approved the study.
